# Systematic Influence of Perceived Grasp Shape on Speech Production

**DOI:** 10.1371/journal.pone.0170221

**Published:** 2017-01-19

**Authors:** Lari Vainio, Aleksi Rantala, Mikko Tiainen, Kaisa Tiippana, Naeem Komeilipoor, Martti Vainio

**Affiliations:** 1 Institute of Behavioural Sciences, Division of Cognitive and Neuropsychology, University of Helsinki, Helsinki, Finland; 2 Institute of Behavioural Sciences, Phonetics and Speech Synthesis Research Group, University of Helsinki, Helsinki, Finland; Leiden University, NETHERLANDS

## Abstract

Previous research has shown that precision and power grip performance is consistently influenced by simultaneous articulation. For example, power grip responses are performed relatively fast with the open-back vowel [a], whereas precision grip responses are performed relatively fast with the close-front vowel [i]. In the present study, the participants were presented with a picture of a hand shaped to the precision or power grip. They were required to pronounce speech sounds according to the front/above perspective of the hand. The results showed that not only the grip performance is affected by simultaneously pronouncing the speech sound but also the production of speech sound can be affected by viewing an image of a grip. The precision grip stimulus triggered relatively rapid production of the front-close vowel [i]. In contrast, the effect related to the power grip stimulus was mostly linked to the vertical dimension of the pronounced vowel since this stimulus triggered relatively rapid production of the back-open vowel [a] and back-mid-open vowel [o] while production of the back-close vowel [u] was not influenced by it. The fact that production of the dorsal consonant [k] or coronal consonant [t] were not influenced by these stimuli suggests that the effect was not associated with a relative front-back tongue shape of the articulation in the absence of changes in any vertical articulatory components. These findings provide evidence for an intimate interaction between certain articulatory gestures and grip types, suggesting that an overlapping visuomotor network operates for planning articulatory gestures and grasp actions.

## Introduction

The present study uses behavioural techniques to investigate interaction between processes that plan articulatory gestures and hand movements. Given that body parts are somatotopically represented in the primary motor cortex [1], at the first glance, this research topic might appear to be rather surprising. However, it is also known that the motor representations of one body part can be involved in planning and executing actions with another body part. In particular, this has been shown to be the case in relation to hand and mouth actions [2,3]. For example, magnetoencephalography (MEG) evidence have shown increased activation in the hand motor area of the primary motor cortex during lip and tongue protrusion task and during silent phoneme production [4]. Correspondingly, electromyographic (EMG) responses of hand muscles are increased by the transcranial magnetic stimulation (TMS) applied to hand motor area if participants are reading, speaking or listening to speech spontaneously during the stimulation [5–7].

In addition, the motor areas outside of the primary motor cortex similarly show overlap in planning articulatory gestures and hand actions. For example, the posterior part of the inferior frontal gyrus, known as Broca’s area, has been shown to be a region where action goals (gesture primitives) of hand and mouth movements are represented [8,9], and therefore it is critical for planning and producing, for example, grasp shapes and articulatory gestures [10]. Broca’s area has been also often associated with anatomical and functional overlap between speech processes and manual processes [11,12].

One reason why there seems to be an interaction between mouth and hand processes in the motor system might be that they are jointly involved in eating behaviour. Indeed, electrical stimulation of certain premotor neurons can trigger ethologically relevant joint hand-mouth behaviour such as closing the hand grip while bringing the hand to the mouth and opening the mouth [13]. A similar effect of electrical stimulation has been recently shown in humans [14]. Moreover, it has been found that the same premotor neurons, called the double grasp neurons, are involved in commanding grasp motor acts with both the mouth and the hand [8]. This evidence was proposed to reflect double grasp preparation processes that are typically accomplished by grasping a piece of food with the hand, bringing the food to the mouth and finally grasping it with the mouth (i.e., taking possession of the food with the mouth).

The mouth-hand mimicry theories [15,16] assume that the overlap between mouth and hand mechanisms may have resulted in a tendency to involuntarily imitate hand actions with articulators (i.e., tongue and lips). In line with these mouth-hand mimicry theories, Darwin already in 1872 noted that people seem to have this kind of tendency as they, for example, often involuntarily clench and unclench their jaws when they are cutting something with scissors [17]. More recently, a similar phenomenon has been observed in young children who perform sympathetic mouth actions, such as tongue protrusions, in imitative synchrony with manual actions [18].

In addition, Gentilucci and his colleagues have provided behavioural evidence for the idea that mouth actions are automatically shaped by simultaneously performed hand actions and correspondingly hand actions are shaped by mouth actions. In these studies, for example, when the participants were required to grasp an object and simultaneously pronounce a meaningless syllable, the more the object required finger opening for the grasp due to its size, the more the participants opened their lips during vocalization [19]. Furthermore, when participants reached to grasp objects while pronouncing an open (/a/) or a close (/i/) vowel, finger opening of the grasp increased with the open vowel in comparison to the close vowel [20].

The recent gestural theories of language evolution [21,22] take this mouth-hand mimicry hypothesis even further and assume that speech might have evolved by transferring communication from hand gestures to articulatory gestures. Likewise, Gentilucci and Corballis [23] have proposed that the most significant evolutionary step in this transfer process might be based on shared programming of hand and mouth grasps in eating behaviour [14] that has boosted transferring communication from hand to mouth, and enabled co-opting grasp-related mouth actions for speech.

If articulators are indeed copying routine hand actions such as grasping, and some articulatory gestures have been co-opted from manual grasp types, it could be assumed that the articulatory gesture repertoire could have been modulated by the pre-existing manual action repertoire. Consequently, it could be hypothesized that some articulatory gestures might be analogues of grasp actions. In support of this hypothesis, interaction between specific mouth gestures and precision grasp actions has been observed in human and non-human apes. Waters and Fouts [24] observed that chimpanzees increasingly produce mouth movements such as protrusion and compression of the lips and tongue during fine manual manipulation, particularly when the manipulation requires the precision grip. Similarly in humans, Higginbotham, Isaak and Domingue [25] observed an increase in electromyographic (EMG) responses of the orbicularis oris muscle—linked to the articulation of bilabial stops and round vowels—during precision grasping. These findings might be viewed as evidence for evolutionary and functional interaction between representations of some communicative mouth gestures and precision grasping.

The articulatory gesture repertoire could be expected to include an oral counterpart not only for the precision grip but also for the power grip if speech mechanisms have indeed evolved on top of the mechanisms that have originally programmed manual actions. In general, the terminal phase of different kinds of grasps can be divided into these two main grip types [26]. The precision grip has evolved in primates for grasping and manipulation of small objects by pinching the object between the tips of the index finger and thumb. In contrast, the power grip has developed for grasping and holding larger objects using a clamp formed by the partly flexed fingers and the palm. These grip types have their very own neural, functional and developmental characteristics [26–28].

Because of the above mentioned identification of two separate grip prototypes, we have recently expanded the investigation of connection between speech and grasping by exploring whether precision and power grips can both be systematically associated with specific articulatory gestures [29]. Our original study provided behavioural support for the view that at least some articulatory gestures may share action planning mechanisms with the precision and power grip. We used a dual-action protocol in which the participants were required to pronounce the syllable displayed on the computer monitor and simultaneously perform either a precision or power grip response according to the colour of the displayed syllable. Power grip responses were linked to relatively short reaction times and small error rates when the pronounced syllable was [kα], [ke] or [hα]. In contrast, precision grip responses were linked to relatively short reaction times and small error rates when the pronounced syllable was [ti], [te] or [hi]. In other words, the consonant [t] and the vowel [i] were associated with the precision grip and the consonant [k] and the vowel [α] were associated with the power grip.

Since the original observation, we have shown this effect in several different task contexts. For example, these speech units facilitate the congruent grip responses when they are read silently or presented auditorily [30]. We have also shown that the effect can be observed in vocal responses: production of these vowels and consonants is facilitated if the participant is required to prepare the grip response prior to onset of the vocal response congruent with the grip [31].

We have proposed that this articulation-grip congruency effect reflects partially overlapped planning of goal shapes of the two distal effectors: a vocal tract shape for articulation and a hand shape for grasping [29–31]. In more detail, we propose that articulation of the consonant [t] is analogous to the precision grip, as in both actions the primary functional component is the tip of the effector (i.e., tip of the tongue and tip of the index finger and the thumb). In contrast, articulation of the consonant [k] involves relatively large surface of the tongue body, which is brought into contact with the soft palate. This articulatory gesture is assumed to be analogous to the power grip, which utilizes the intermediate and proximal components of all fingers as well as the palmar surface of the hand. In fact, similar analogy between the power grip and the dorsal consonants has been proposed also by Ramachandran and Hubbard [32] who suggested that hand actions that involve flexion of fingers toward palmar crease, as is the case with the power grasp, is mimicked by articulators in the gestures in which tongue body is brought onto back of the palate.

Moreover, we propose that articulation of the vowel [i] is associated with the precision grip because the aperture of different parts of a vocal tract associated with this articulation remains relatively closed in the same way as the aperture between the index finger and the thumb remains relatively small in precision grasping as it is typically used to grasp small objects. In the same vein, articulation of the vowel [α] is associated with the power grip because articulation of that vowel requires relatively large aperture of the vocal tract in the same way as the aperture between the thumb and rest of the fingers is spread relatively wide in power grasping as it is typically used to grasp large objects.

In sum, we propose that an innate overlap in mechanisms that are involved in planning mouth and hand actions might have led, in turn, to the involuntary tendency to imitate routine hand actions with articulators leading also, at the evolutionary scale, to the utilization and development of articulatory gestures that bear at least crude relation to the hand gestures that they accompany. In other words, development of some articulatory gestures may have been boosted by partially overlapped planning mechanisms between certain mouth actions and precision and power grasp actions. Consequently, we assume that the current repertoire of articulatory gestures includes ones that are linked with the precision grasp and others that are linked to the power grasp.

### Perception of grasping modulates grasp-planning and speech processes

Because the primary purpose of the present paper is to explore how grasp-related information of perceived stimuli influences speech performance, it is important to consider how grasp-related information of perceived stimuli is processed in the motor planning system in general. Firstly, the grasp motor processes can be automatically modulated by perceived grasping [33,34]. The phenomenon in which motor processes are modulated by action-based stimuli has been linked to the so-called mirror mechanisms, which are responsible for encoding observed actions in the motor representations, but which are also involved in programming the corresponding actions [35,36]. These mirror neurons were initially discovered in the premotor region F5 of monkey. Subsequently, for example, brain imaging [37] and single cell recording [38] studies have shown that a similar mirror neuron system can be also observed in humans.

Regarding grasp actions, some mirror neurons in the premotor region F5 encode actions in a strictly congruent manner [39]. In other words, perceiving precision grasping activates the motor representation that is involved in producing the precision grasp whereas perceiving power grasping activates the motor representation that is involved in producing the power grasp. Furthermore, although these hand motor representations were originally assumed to preferably operate for perceiving object-directed grasp actions [40], perceiving mimicked grasp actions have also been observed to activate these motor areas [41].

Importantly for our current methodological purposes, the interaction between perceived grasping and grasp performance has also been investigated using a behavioural technique [42]. In that study, the participants were presented with stimuli in which a hand was making the precision or power grasp in absence of any goal object. The stimuli consisted of two different kinds of precision and power grasp stimuli. The final shape of the grasps was either conventional or unconventional. In the unconventional stimuli of the precision and power grip, the final position of the thumb was slightly twisted from its typically observed position. The participants were required to perform precision and power grip responses according to the categories of conventional and unconventional grip type. The main finding was that precision and power grip responses were made relatively rapidly and accurately when the hand stimuli presented conventional precision and power grasps, respectively. The effect was proposed to reflect automatic activation of a specific grasp motor program triggered by the observed grip type.

Perceived grasping not only influences the hand motor processes but it can also modulate the mouth motor processes. For example, some mouth mirror neurons in monkeys are activated by observation of hand and mouth movements as long as they have the same goal [33]. Similarly in humans, articulation is systematically influenced by observed grasp actions. For example, lip opening increases when the participants are asked to vocalize the syllable [ba] and simultaneously observe a hand which is grasping a large object, using the power grasp, as opposed to a small object, using the precision grasp [43,44]. However, this effect has not been reported in relation to observing a mimicked grasp action in absence of a goal object. Moreover, although the stimuli of that study consisted of a dynamic reach-to-grasp action, the vocal response was performed in the terminal phase when the hand was holding the goal object by the precision or power grip. Consequently, it is not known whether the effect is based on the dynamic properties of the observed reach-to-grasp action (e.g., increased finger opening during reach phase of the prehension) or whether simply perceiving static image of a hand shaped to the precision or power grip, in the absence of any goal object, could influence vocal responses. Indeed, if the articulatory and grasp processes are connected, one might assume that observing an image of the precision or power grip–even in the absence of any goal object–might produce a corresponding effect on vocal responses that has been previously reported in relation to manual responses by Vainio et al. [42]. Finally, in those studies reported by Gentilucci and his colleagues, the participants were pronouncing the same syllable (i.e., [ba]) in all experimental trials; the effect reflected the extent to which the observed grasping influenced lip opening related to vocalization of this syllable. However, it is not known whether selection of specific articulatory gestures could be biased by perceiving a hand that is shaped to the precision or power grip. As a consequence, the present study investigated whether presenting an image of the precision grip to the participants would prime vocalization of those speech units that are hypothetically congruent with that grip type (e.g., [t] and [i]) and whether presenting an image of the power grip to the participants would prime vocalization of those speech units that are hypothetically congruent with that grip type (e.g., [k] and [α]).

## Experiment 1

In the paradigm used by Vainio et al. [42] the participants were required to respond using the precision or power grip while they were presented with the precision or power grasp stimuli. In the current task, the manual responses were replaced by vocal responses. The participants were presented with an image of the precision or power grip and they were required to vocalize as fast as possible one of the two vocal response choices (Block 1: [i] or [α]; Block 2: [ti] or [kα]) according to whether the hand stimulus was shown from the front or above perspective. Similarly to the traditional stimulus-response compatibility (SRC) tasks [45], we were interested in measuring how a task-irrelevant stimulus property (i.e., a grip type) influences responses. We assumed that in this task, articulatory motor responses would be primed by visual properties in the same way as grasp motor responses are primed in the task reported by Vainio et al. [42]. That is, the perceived grip type could result in automatic activation of the articulatory gesture associated with the grip type leading to relatively rapid production of this articulatory gesture in comparison to the opposite response. Based on our previous observations of motor interactions [29], we predicted that the visual precision grip stimuli would be associated with relatively rapid vocal responses of [i] and [ti] whereas the visual power grip stimuli would be associated with relatively rapid vocal responses of [α] and [kα].

In addition to observing whether the interaction between grip types and speech units would manifest themselves in reaction times, we also anticipated that the perceived grip type might have some systematic influence on certain elements of vocal sounds. Consequently, the present study also explored whether intensity, fundamental frequency (*f*_*0*_), formant 1 (F1) or formant 2 (F2) are modulated by the viewed grip type. Changes in F1 reflect changes in the vertical dimension of the shape of the oral cavity during articulation (i.e., F1 increases with mouth opening) while changes in F2 reflect changes in the horizontal dimension of the tongue shape during articulation (i.e., F2 increases as the tongue is pushed more forward) [46]. As a consequence, given that we have previously proposed that front-close articulations are connected to precision grasp processes whereas back-open articulations are connected to power grasp processes [29], we anticipated that viewing the precision grip might decrease F1 values and increase F2 values, while the power grip might increase F1 values and decrease F2 values. Moreover, intensity and *f*_0_ have been shown to change as a function of changes in F1 [47,48], and consequently we might also observe some grip-triggered modulations in these elements of vocal sounds.

### Methods

#### Participants

Sixteen naïve volunteers participated in Experiment 1 (20–38 years of age; mean age = 24.8 years; 4 males; 1 left-handed). All participants were native speakers of Finnish and reported normal hearing and normal or corrected-to-normal vision. We obtained a written informed consent from all participants. The study was conducted according to the principles expressed in the Declaration of Helsinki. The study was approved by the Ethical Review Board in Humanities and Social and Behavioural Sciences at the University of Helsinki.

#### Apparatus, stimuli and procedure

**Fig 1:**
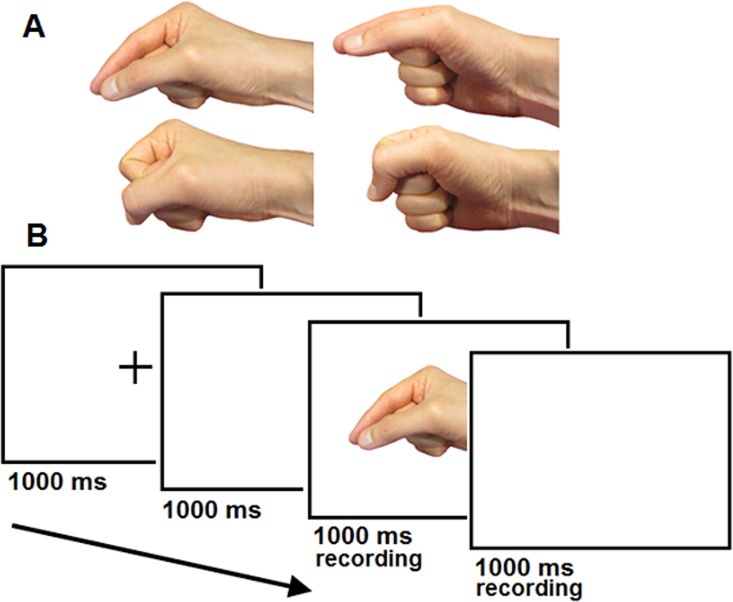
**The illustration of the stimuli (A) and the design (B) used in Experiments 1 and 2.** The participants were required to perform a vocal response (e.g., [i] or [α]) according to the perspective of the hand (above-perspectives of the precision and power grip are presented on the left side and front-perspectives of these grips are presented on the right side).

Each participant sat in a dimly lit room with his or her head 60 cm in front of a 19-in. CRT monitor (screen refresh rate: 85 Hz; screen resolution: 1280 x 1024). A head-mounted microphone was adjusted close to the participant’s mouth. The target stimuli (Fig 1A) consisted of four different still images of a hand. The hand images were always pointing from right to left, and they were presented in colour. Two images presented a hand in a precision grip position and two presented a hand in a power grip position. In addition, both of these grip types were presented in two different perspectives: front-perspective and above-perspective. Hence, the following images were used as stimuli: precision grip/above (horizontally 12.4° x vertically 7.2°), precision grip/front (12.4° x 7.2°), power grip/above (10.9° x 6.2°) and power grip/front (10.9° x 6.2°). The stimuli were presented centrally on the monitor on a white background.

The experiment began with practice trials. Each participant was given as much practice as it took to perform the task fluently. The trial started with a presentation of a central fixation cross (1° x 1°), which was displayed for 1000 ms. A blank screen was displayed for 1000 ms after the offset of the cross. Then the target was presented for 1000 ms. Target stimuli were presented in randomized order. Finally, a blank screen was displayed for 1000 ms after the offset of the target. The illustration of this design is presented in Fig 1B.

The experiment was divided into two blocks. The participants were allowed to have a short break between the blocks and within each block. In the vowel block, the participants were asked to respond by pronouncing either the vowel [i] or [α], and in the syllable block, they were asked to respond by pronouncing either the syllable [ti] or [kα]. The order of these blocks was counterbalanced between the participants. In both blocks, the participants were required to respond as fast and accurately as possible according to the perspective of the hand. Half of the participants responded in the vowel block by pronouncing [i] to the above-perspective and [α] to the front-perspective, and in the syllable block by pronouncing [ti] to the above-perspective and [kα] to the front-perspective. Other half responded in opposite response-to-perspective mapping. In total, the experiment consisted of 240 trials [30 repetitions x 2 (grip) x 2 (response) x 2 (block)].

The vocal responses were recorded for 2000 ms starting from the onset of the target object. At the beginning of the experiment, the recording levels were calibrated individually using the voice calibration function of Presentation® software (Version 16.1, www.neurobs.com) so that the recording levels would match with the natural intensity of the participant’s voice. In the calibration, the participants were required to pronounce the vowels [i] and [α] one after another approximately once every second. Stimulus presentation and sound recording were done with Presentation® software.

### Results

Reaction times: Vocal data was analysed using Praat v. 5.3.49 (http://www.praat.org). Onsets of vocalization were located individually for each trial as the first observable peak in the acoustic signal for the consonant burst (consonant block) or the vowel (vowel block). Reaction times were measured from the onset of the target object to the onset of the vocalization. Errors (i.e., the participant uttered the wrong speech unit) and RTs more than two standard deviations from each participant’s condition means were excluded from the reaction time analysis. Of the trials, 0.7% were removed as errors and 4.2% were removed as outliers. The combined removal of errors and outliers left 95.1% of the raw data as correct responses. In addition, six trials were missing from recordings for an unknown technical reason. The condition means of this remaining data were computed for each participant and subjected to a repeated-measures analysis of variance (ANOVA) with the within-subjects variables of Grip (precision or power) and Response ([i]/[ti] or [α]/[kα]), and Block ([i]-[α] or [ti]-[kα]). Post hoc comparisons were performed by means of t-tests applying a Bonferroni correction when appropriate. A partial-eta-squared statistic served as an effect size estimate.

**Fig 2:**
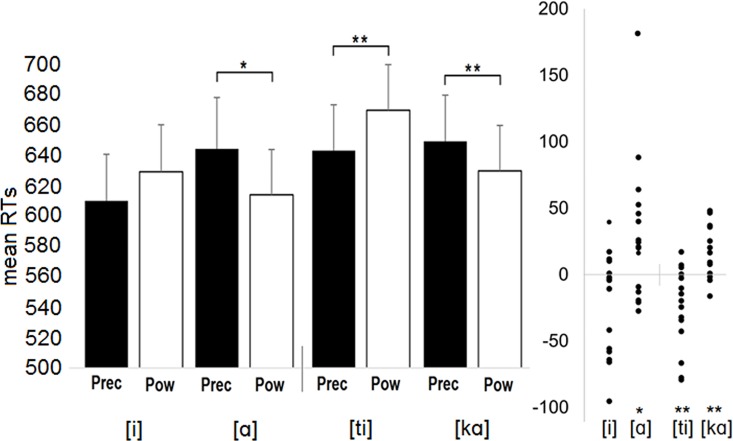
The mean reaction times (RTs) for Experiment 1 as a function of the stimulus type (Grip) and the pronounced speech unit (Response) (Block 1: [i] vs [α]; Block 2: [ti] vs [kα]). RTs were faster when the pronounced speech unit was congruent rather than incongruent with the stimulus type (e.g., [kα]-power grip). The right section of the figure presents the distribution of the mean RT difference between Grips (Mean_Precision_—Mean_Power_) for four response categories. Each data point shows mean for a single participant. In those data points that fall below the zero line, mean RTs are faster with the precision grip stimulus, and in those that rise above the zero line, mean RTs are faster with the power grip stimulus. The vertical line on the zero axis separates one block from another. * < .05, ** = p < .01, *** = p < .001.

The participants made only 27 errors altogether. Consequently, the error data was not analysed. The analysis of reaction times revealed a significant interaction between Grip and Response, *F*(1,15) = 19.39, *MSE* = 17510.13, *p* = .001, *η*_*p*_^*2*^ = .564. This interaction is presented in Fig 2. The three-way interaction between Grip, Response and Block was not significant (*p* = .798). In the vowel block, though the vowel [i] was produced, on average, faster when the visual stimulus presented the precision grip (M = 609 ms) rather than the power grip (M = 628 ms), this effect was not statistically significant (p = .065). In contrast, the vowel [α] was produced faster when the visual stimulus presented the power grip (M = 613 ms) rather than the precision grip (M = 643 ms) (p = .044). In the syllable block, the syllable [ti] was produced faster when the stimuli were presenting the precision grip (M = 640 ms) rather than the power grip (M = 666 ms) (p = .003). In contrast, the syllable [kα] was produced faster when the stimuli were presenting the power grip (M = 627 ms) rather than the precision grip (M = 646 ms) (p = .003). Finally, when the mapping condition (MP1: [i]/[ti]-above vs. [α]/[kα]-front; MP2: [i]/[ti]-front vs. [α]/[kα]-above) was included in the analysis as a between-subjects factor, the three-way interaction between Grip, Response and Mapping (*p* = .828) or the four-way interaction between Grip, Response, Block and Mapping (*p* = .606) were not significant.

Voice characteristics: For the intensity value, the peak intensity of the voiced vowel section (the section between the onset and offset of the vowel vocalization) was selected. The *f*_*0*_ and formants F1 and F2 were all calculated as a median value from the middle third of the vowel section of the vocalization area. In few occasions, the formant value that was provided as an output of the Praat analysis clearly exceeded variations that can be normally observed within voice characteristics of the given vowels. For example, the Praat might have in few cases mixed F2 values with F3 values. These values (0.3%) were removed from the analysis. Then the values more than two standard deviations from each participant’s condition means of each vocal response condition for intensity (0.2%), *f*_*0*_ (0.3%), F1 (0.5%) and F2 (0.2%) were excluded from the voice characteristic analysis.

The analysis of intensity did not reveal any significant main effects or interactions. Furthermore, the analysis did not reveal any main effects or interactions related to *f*_*0*_, F1 or F2 that would have been relevant to our hypothesis. However, as could be expected, these spectral components were modulated as a function of a pronounced vowel. That is, the analysis of *f*_*0*_, F1 and F2 revealed a significant main effect of Response. *F*_*0*_ was lower for Responses [α]/[kα] (M = 180 Hz) than for [i]/[ti] (M = 200 Hz) (p < .001). Similarly, F2 was lower for Responses [α]/[kα] (M = 1115 Hz) than for [i]/[ti] (M = 2633 Hz) (p < .001). In contrast, F1 was lower for Responses [i]/[ti] (M = 333 Hz) than for [α]/[kα] (M = 591 Hz) (p < .001).

### Discussion

The results of Experiment 1 showed that [ti] vocalizations were performed relatively rapidly if the viewed hand was shaped to the precision grip whereas [α] and [kα] vocalizations were performed relatively rapidly if the viewed hand was shaped to the power grip. In addition, [i] vocalizations showed a congruent pattern with the precision grip albeit this effect was not statistically significant. These findings supports our hypothesis according to which an overlapping visuomotor network operates for perceiving and planning articulatory gestures and grasp actions, and that there are systematic interactions between the precision and power grip representations and specific articulatory gestures within this network. For example, [α] articulations are not only associated with power grip performance [27] but also observing the image of the power grip can similarly facilitate [α] articulations.

## Experiment 2

The results of Experiment 1 leave open couple of questions: Firstly, they do not conclusively show whether viewing the precision or power grip is associated with facilitated pronunciation of consonants [t] and [k], respectively. That is, because the effect is mostly linked to pronouncing the vowels alone, it remains unclear whether the consonant content contributed to the effect in the [ti]-[kα] block. As a consequence, Experiment 2 investigates whether the effect can be associated with pronouncing the consonants [t] and [k] by asking the participants to pronounce the syllables [te] or [ke] in the task used in Experiment 1. The consonants were coupled with the vowel [e] because it is difficult to pronounce them alone. The vowel [e] was selected because it is unrounded and relatively neutral in Finnish language in terms of tongue position.

Secondly, given that [α] is an open-back vowel, it also remains unresolved whether the effect reflects articulatory processes related to vertical or horizontal dimension of the gesture. Consequently, in addition to the [te]-[ke] block, Experiment 2 also included the blocks [i]-[o] and [i]-[u] in order to investigate this matter. If the power grip is linked to facilitated [o] responses instead of [u] responses, it would suggest that the effect reflects articulatory processes related to vertical dimension of the gesture because [o] and [u] are both back vowels but [o] is mid-open while [u] is closed. In contrast, if the power grip is linked to facilitated [o] and [u] responses, it would suggest that the openness of the vocalization does not contribute to the effect but it is exclusively based on articulatory processes related to the horizontal dimension.

### Methods

#### Participants

Eighteen naïve volunteers participated in Experiment 2 (20–30 years of age; mean age = 24.8 years; 2 males; 1 left-handed). All participants were native speakers of Finnish and had a normal or corrected- to-normal vision. We obtained a written informed consent from all participants. The study was conducted according to the principles expressed in the Declaration of Helsinki. The study was approved by the Ethical Review Board in Humanities and Social and Behavioural Sciences at the University of Helsinki.

#### Apparatus, stimuli and procedure

The apparatus, environmental conditions, calibration, stimuli, procedure and task were the same as those in Experiment 1. However, in contrast to Experiment 1, this experiment consisted of three blocks in which the participants were required to provide different vocal responses. Depending on the block, the participants had to pronounce the consonants [te] or [ke], the vowels [i] or [o], or the vowels [i] or [u]. The order of the blocks was counterbalanced. Hence, in total, the experiment consisted of 360 trials [30 repetitions x 2 (grip) x 2 (response) x 3 (block)].

### Results

Reaction times: Reaction times and errors were processed similarly as in Experiment 1. Of the trials, 1.4% were removed as errors and 4.7% were removed as outliers. The combined removal of errors and outliers left 94.9% of the raw data as correct responses. The condition means of this remaining data were computed for each participant and subjected to a repeated-measures analysis of variance (ANOVA) with the within-subjects variables of Grip (precision or power) and Response ([te]/[i]/[i] or or [ke]/[o]/[u]), and Block ([te]-[ke], [i]-[o] or [i]-[u]). Post hoc comparisons were performed by means of t-tests applying a Bonferroni correction when appropriate. A partial-eta-squared statistic served as an effect size estimate.

**Fig 3:**
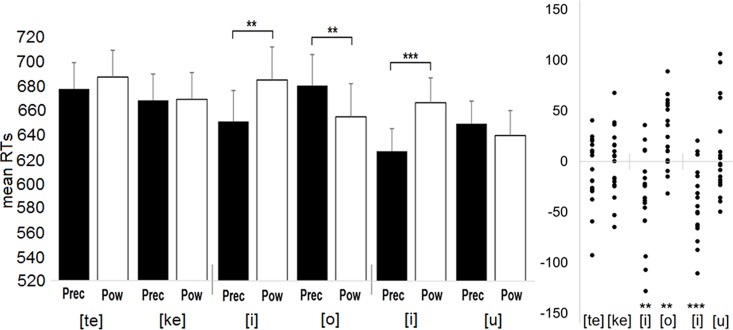
The mean reaction times (RTs) for Experiment 2 as a function of the stimulus type and the pronounced speech unit (Block 1: [te] vs [ke]; Block 2: [i] vs [o]; Block 3: [i] vs [u]). RTs were faster when the pronounced speech unit was congruent rather than incongruent with the stimulus type (e.g., [i]-precision grip). The right section of the figure presents the distribution of the mean RT difference between Grips (Mean_Precision_—Mean_Power_) for six response categories. Each data point shows mean for a single participant. In those data points that fall below the zero line, mean RTs are faster with the precision grip stimulus, and in those that rise above the zero line, mean RTs are faster with the power grip stimulus. The vertical line on the zero axis separates one block from another. * < .05, ** = p < .01, *** = p < .001.

The participants made only 93 errors altogether. Due to the low error rate, the error data was not analysed. The analysis of reaction times revealed a significant interaction between Grip and Response, *F*(1,17) = 21.63, *MSE* = 20995.08, *p* < .001, *η*_*p*_^*2*^ = .560. The three-way interaction between Grip, Response and Block was marginally significant, *F*(2,34) = 3.21, *MSE* = 3235.89, *p* = .053, *η*_*p*_^*2*^ = .159. This interaction is presented in Fig 3. In the [te]-[ke] block, the grip type did not influence vocal responses ([te]: p = .228; [ke]: p = .921). However, in the [i]-[o] block, the vowel [i] was produced faster when the stimuli were presenting the precision grip (M = 650 ms) rather than the power grip (M = 684 ms) (p = .009). In contrast, the vowel [o] was produced faster when the stimuli were presenting the power grip (M = 654 ms) rather than the precision grip (M = 680 ms) (p = .004). Similarly, in the [i]-[u] block, the vowel [i] was produced faster when the stimuli were presenting the precision grip (M = 627 ms) rather than the power grip (M = 667 ms) (p < .001). However, the pronunciation speed related to the vowel [u] was not significantly influenced by the grip type in that block (p = .377). Finally, when the mapping condition (MP1: [te]/[i]/[i]-above vs. [ke]/[o]/[u]-front; MP2: [te]/[i]/[i]-front vs. [ke]/[o]/[u]-above) was included in the analysis as a between-subjects factor, the three-way interaction between Grip, Response and Mapping (*p* = .202) or the four-way interaction between Grip, Response, Block and Mapping (*p* = .303) was not significant.

Finally, in order to explore whether the congruency effect changes over time between the early and late responses within a single experimental block, we carried out a separate repeated-measures ANOVA by including a factor of Trial position to the analysis: The first third (early), the second third (middle), and the final third (late). Because cutting the data set into three parts decreased the statistical power of analysis in catching the interaction effect between Grip and Response in separate levels of Trial position variable, the variables of Grip and Response were replaced by a new factor of Grip-Response congruency. In this way, we increased the number of data points that reflected the congruency between Gip and Response, the most important variable of this analysis. The congruent conditions were those where the presented grip was the precision and the vocal response was [i] and where the presented grip was the power and the vocal response was [o]. In contrast, the incongruent data points included those cases where the presented grip was the precision and the vocal response was [o] and where the presented grip was the power and the vocal response was [i]. As a result, the analysis included the variables of Trial position (early, middle or late) and Grip-Response congruency (congruent or incongruent). This analysis was carried out only for the data of the block 2 ([i]-[o]) because in that block the effect was significant for both vocal responses.

The analysis showed that reaction times did not differ between the early, middle and late responses, F(2,34) = 0.17, MSE = 1924.66, p = .841, *η*_*p*_^*2*^ = .010. As expected, the main effect of Grip-Response congruency was significant, F(1,17) = 19.20, MSE = 19655.94, p < .001, *η*_*p*_^*2*^ = .530. The vowels were pronounced faster in congruent (M = 661 ms) rather than in incongruent conditions (M = 688 ms). Importantly, the interaction between Trial position and Grip-Response congruency was not significant [F(2,34) = 0.27, MSE = 258.95, p < .765, *η*_*p*_^*2*^ = .016] showing that the congruency effect did not change over time between the early, middle and late responses. That is, the congruency effect between the perceived grip type and the vocal response was observed with the early (congruent: M = 655 ms; incongruent: M = 679 ms; p = .038), middle (congruent: M = 659 ms; incongruent: M = 692 ms; p = .008) and late (congruent: M = 670 ms; incongruent: M = 694 ms; p = .025) responses.

Voice characteristics: Voice characteristics were analysed in the same way as in Experiment 1. Firstly, the values (0.2%) that clearly exceeded variations that can be normally observed within voice characteristics of the given vowels were removed from the analysis. Then the values more than two standard deviations from each participant’s condition means of vocal response condition for intensity (0.1%), *f*_*0*_ (0.6%), F1 (0.3%) and F2 (0.3%) were excluded from the voice characteristic analysis.

The analysis of intensity did not reveal any significant main effects or interactions. Furthermore, the analysis did not reveal any main effects or interactions related to *f*_*0*_, F1 or F2 that would have been relevant to our hypothesis. However, as could be expected, these spectral components were modulated as a function of a pronounced speech unit. That is, the analysis of *f*_*0*_, F1 and F2 revealed a significant interaction between Response and Block. In the [i]-[o] block, *f*_*0*_ was significantly lower for [o] (M = 194 Hz) than for [i] (M = 208 Hz) (p = .001). Similar difference was not observed in the [te]-[ke] (p = .176) or [i]-[u] (p = .225) block. Regarding F1, in the [te]-[ke] block, F1 was lower for [ke] (M = 535 Hz) than for [te] (M = 552 Hz) (p < .001). In the [i]-[o] block, it was lower for [i] (M = 336 Hz) than for [o] (M = 481 Hz) (p < .001). Similarly, in the [i]-[u] block, it was lower for [i] (M = 342 Hz) than for [u] (M = 360 Hz) (p = .012). Regarding F2, in the [i]-[o] block, F2 was lower for [o] (M = 888 Hz) than for [ti] (M = 2749 Hz) (p < .001). Similarly, in the [i]-[u] block, it was lower for [u] (M = 665 Hz) than for [i] (M = 2735 Hz) (p < .001). There was not observed any significant difference in the [te]-[ke] block (p = .509) in F2 values.

### Discussion

The results of Experiment 2 expand the findings of Experiment 1. Firstly, Experiment 2 suggest that the articulation-grip congruency effect cannot be triggered by the consonants [t] or [k] alone. Secondly, Experiment 2 shows that the openness dimension of articulation contributes to the articulation-grip congruency effect. That is because, the power grip was associated with facilitated [o] vocalizations in the [i]-[o] block, while it was not associated with facilitated [u] vocalizations in the [i]-[u] block even though it is also a back vowel as is the vowel [o] but the difference is that [u] is a closed vowel.

## General Discussion

It has been previously found that when participants pronounce a syllable and simultaneously perform either a precision or power grip response, the manual responses are made faster when there is a congruency between the syllable and the grip response (e.g., [ti]-precision grip) [29]. The current study shows that this articulation-grip congruency effect can be also observed in reaction times of vocal responses when the participants are presented with a hand that is shaped to the precision or power grip. These findings support the account that the overlapping visuomotor network operates for perceiving and planning articulatory gestures and grasp actions, and there are systematic interactions between the precision and power grip representations and specific articulatory gestures within this network. That is, the interaction between certain articulatory and manual grasp processes manifest itself not only in behavioural level when articulation and grasping has to be performed simultaneously, but also viewing an image of a hand grip similarly primes articulatory processes that are congruent with the perceived grip type.

In more detail, the results of Experiment 1 demonstrated that the perceived grip type has consistent impact on vocal responses. Reaction times of those vocal responses that have previously been shown to be associated with precision grip performance (i.e., articulation of the syllable [ti]) were faster when the perceived stimulus was a hand shaped to the precision grip. Similar pattern was observed between the precision grip and the vowel [i], albeit this effect was not statistically significant. In contrast, reaction times of those vocal responses that have previously been shown to be associated with power grip performance (i.e., articulation of the syllable [kα] or the vowel [α]) were faster when the perceived stimulus was a hand shaped to the power grip.

The results of Experiment 2 showed that the effect of the perceived grip type on vocalization is not observed with the syllables [te] and [ke]. That is, when only the consonant content of the articulation changes between alternative vocal responses, in absence of changes in the vowel content, the effect was removed. However, given that in Experiment 1 the congruency effect with the vowel [i] was absent whereas with the syllable [ti] the effect was clear, it might be speculated that the consonant [t] nevertheless contributed to the effect; At least when it was pronounced in the consonant-vowel (CV) context in which the vowel production, similarly to the consonant [t], required pushing the tongue into a high-anterior position. In fact, the frame/content theory of evolution of speech production [49] assumes that CV syllables form the central units of speech that evolved from lip- and tongue-smack types of vocalizations. In this theory, the consonant content of the vocalization reflects, or evolved from, the closing phase of the lip- and/or tongue-smack, while the vowel content is associated with the opening phase of these orofacial gestures. According to the theory, the fact that the front vowels are preferentially associated with coronal consonants while the back vowels are preferentially associated with dorsal consonants [50] is a reflection of these above-mentioned milestones of speech evolution. Consequently, in the context of the frame/content theory, it could be assumed that the motor representations of the precision and power grip optimally interact with the articulatory representations of CV syllables. This concerns particularly those syllables where the coronal consonant [t] is associated with the front vowel [i] (i.e., [ti]) or where the dorsal consonant [k] is associated with the back vowel [α] (i.e., [kα]). According to this view, the interaction might be related to articulatory representations of individual consonants or vowels only subordinately. This might explain why the effect was observed with the syllable [ti] (Experiment 1) while it was missing with the vowel [i] (Experiment 1) and with the syllable [te] (Experiment 2). Nevertheless, the results of Experiment 2 ([i]-[o] block & [i]-[u] block) also showed that the effect can be observed with the vowel [i] even when it is not pronounced in the CV context. This contradicts with the idea that the effect operates ideally with articulatory representations of CV syllables. Consequently, the idea that this effect works optimally with CV syllables that are build from a coronal consonant and a front vowel (or alternatively from a dorsal consonant and a back vowel) warrants for further investigation.

It appears that two criteria have to be met so that the vocalization can be facilitated by the perceived precision grip. Firstly, the articulation has to be performed with a narrow front cavity in front of the tongue constriction (i.e., the tongue blade has to be pushed into a high-anterior position) as is the case with front vowels. Secondly, in the articulation, the jaw has to be kept relatively closed as is the case with close vowels. The vowel [i] meets both of these criteria and consequently shows the effect with the precision grip. Furthermore, the results suggest that two criteria have to be also met so that the vocalization can be facilitated by the perceived power grip. Firstly, the articulation has to be performed with a large front cavity so that the tongue is considerably retracted as is the case with back vowels. Secondly, the jaw has to be lowered as is the case with open vowels. The vowel [α] clearly meets both of these criteria and consequently shows the effect with the power grip. It has to be mentioned that in Finnish, the jaw and tongue are not just slightly lower in [o] than in [u], but the tongue is also slightly more retracted in [o] than in [u] [51]. Therefore, [o] marginally meets the openness criterion and clearly meets the backness criterion for being associated with the power grip. Consequently, it shows the effect with the power grip. However, the vowel [u] does not meet the frontness criterion so that it could be associated with the precision grip, but it neither meets the openness criterion so that it could be associated with the power grip. Consequently, it is not associated with either of the grip types.

The present study did not reveal any grip-related modulations of voice spectra components. Regarding the opening of vocal organs reflected by F1 values, for example, the syllable [α] appeared to be articulated in the same manner regardless of whether the perceived hand was shaped to the precision or power grip. In contrast, Gentilucci et al. [43,44] found that the power grasp stimuli were also linked to increased F1 values that supposedly reflected increased opening of the vocal tract as a function of the grasp type. This discrepancy might be caused by methodological differences. The behavioural effects observed in the current study are likely to reflect relatively coarse stimulus triggered biases in selection of the vocal response. For example, observed power grip stimulus might activate both the stored prototypical representation of the power grip and the corresponding articulatory gesture (e.g., [o]) which consequently results in decreased reaction times of [o] responses in comparison to [i] responses. In contrast, the effects observed by Gentilucci and his colleagues [43,44] are more likely to reflect stimulus-triggered modulations of actual vocal performance as their participants were not required to select between vocal response alternatives; they were required to pronounce the same syllable in each trial.

In our original study, the precision and power grip responses were facilitated by simultaneously pronouncing the consonant [t] or [k], respectively [29]. In addition, our recent observations [31] have shown that the articulation-grip congruency effect does not only manifest itself in manual reaction times, but vocal reaction times can also be systematically modulated as a function of performed grip types. That is, the consonant [t] is vocalized relatively fast when it is executed together with precision grip while [k] is vocalized relatively fast when it is executed together with power grip. However, the present study did not replicate this articulation-grip congruency effect with the consonants [t] and [k]. The present data do not provide a conclusive explanation of this discrepancy. However, it is noteworthy that the presently observed visually triggered congruency effect operates mostly within the vertical dimension of vowel articulation, related to differential shaping of an oral cavity involving jaw opening, which in turn provides clear visual cues about the articulated vowel. This characteristic might make processes related to production of these kinds of vowels relatively sensitive to specific visual cues such as vertical mouth movements of other individuals or different grasp types. People have learned to associate these kinds of vowels with specific visual cues. In contrast, the previously observed effect related to [t] and [k] operates within the horizontal dimension of consonant articulation. Thus, as these consonants are mostly produced by shaping the tongue inside the mouth, access to visual features related to these different consonants is mostly removed. This might be the reason why processes related to production of these kinds of consonants are not similarly sensitive to specific visual cues. People have not learned to associate them with any visual cues. It is possible that influence of grasp-related information on production of the consonants [t] and [k] might require that the grasp is actually performed by a participant him/herself by, for example, squeezing an object with the precision or power grip.

### Theoretical considerations related to the interaction between the grip types and the production of different vowels

Similarly to the original study [29], the present study also explored the interaction between processes that plan certain articulatory gestures and the terminal phase of the precision and power grasp actions. However, it is noteworthy that even though in the terminal phase of the precision and power grasp the hand is closed, we have nevertheless proposed that the effect, regarding to the vowel production, mostly reflects analogy between the specific grip type and openness/closeness dimension of the vowel production. That is, the precision grip is associated with the closed vowel and the power grip is associated with the open vowel. Hence, one might rightly question the proposed analogy between these grip types and the articulatory gestures required for producing these vowels because the terminal phase of the power grip is equally close as the terminal phase of the precision grip.

The effect has to be placed within a wider context of sensory-motor planning processes so that our view concerning the effect can be comprehended. Regarding manual grasping, our view is related to some extent to the mirror neuron model of grasp development [52]. According to this model, infants first learn to use grasp affordance information of the object (e.g., size) to program grasping. At this stage, children learn to associate small objects with the precision grip and with the relatively narrow finger opening whereas large objects are associated with the power grip and with the relatively wide finger opening. Gradually they also learn to relate this grasp affordance information of objects to corresponding visual hand information by observing their own hand while grasping objects. Later, this ability is extended to cover mirroring of grasp actions of other individuals.

The critical parts of this grasp development model [52] are in line with behavioural evidence. A relatively large object automatically facilitates selection of the power grip response when it is perceived [53], and increases finger opening when it is reached to grasp [54]. The opposite occurs in relation to relatively small objects: they are associated with facilitated selection of the precision grip response and relatively narrow finger opening [53,54]. Moreover, corresponding effects can be observed even with semantic size information. For example, the magnitude of a number has been shown to influence grip selection processes as well as finger opening; small numbers are associated with the precision grip and narrow finger opening whereas large number are associated with the power grip and wide finger opening [55–57].

Taken together, the grasp development model [52] as well as related behavioural evidence [53–57] assumes that grasp planning is programmed within the associative sensory-motor network that integrates object size information with the relevant grasp parameters including the grip type and the level of finger opening. According to this view, it could be predicted that, for example, solely seeing a large object or number as well as the transport (i.e., a wide finger opening) or the terminal phase (i.e., the power grip) of the power grasp prehension all activate overlapping sensory-motor network related to planning processes of the power grasp. The objective of the present paper is to place also the articulatory processes within this framework. We propose that mechanisms that are responsible for planning certain articulatory gestures also operate within this network leading to facilitated selection of articulatory gesture for producing an open vowel when the power grip is viewed and a close vowel when the precision grip is viewed. This account also predicts that similar modulation of articulatory processes should be observed if participants are presented with a small vs. large sized object or a number with a small vs. large magnitude. For example, seeing a large number, in comparison to a small number, should facilitate selection of an open vowel, in comparison to a close vowel. These predictions should be investigated in future in order to validate our hypothesis.

## Conclusion

The present study shows that not only are manual grasp responses influenced by observed grasp actions [40] but similar SRC effects can also be observed in relation to vocal responses. Reaction times of those vocal responses that are associated with precision grip execution (articulation of the vowel [i]) are relatively short when participants see a hand shaped to the precision grip. In contrast, reaction times of those responses that are associated with the power grip execution (e.g., articulation of the vowel [α]) are relatively short if the seen stimulus is a hand shaped to the power grip. The effect appears to be mostly linked to processes responsible for programming vertical mouth movements related to vowel production. In addition to showing evidence that there is intimate and systematic interaction between certain articulatory gestures and grip types, our results also suggest that the overlapping visuomotor network operates for planning articulatory gestures and grasp actions as well as perceiving grasp actions.

## Supporting Information

S1 Table Datasets for Experiment 1 used for statistical analyses. (XLSX)

S2 Table Datasets for Experiment 2 used for statistical analyses. (XLSX)
